# Maternal, neonatal, and nutritional risk factors for medical and surgical necrotizing enterocolitis

**DOI:** 10.1038/s41372-024-02066-3

**Published:** 2024-07-19

**Authors:** Clare Essex, Clifford Hegedus, Katherine Vincent, Alanna Shiflett, Allison Rohrer, Katherine E. Chetta

**Affiliations:** 1 Medical University of South Carolina, 96 Jonathan Lucas Street Suite 601, MSC 617, Charleston, SC 29425 USA; 2 C.P. Darby Department of Pediatrics, Division of Neonatal-Perinatal Medicine, Medical University of South Carolina, 10 McClennan Banks Drive, MSC 915, Charleston, SC 29425 USA; 3 C.P. Darby Children’s Research Institute, Medical University of South Carolina, Shawn Jenkins Children’s Hospital, 171 Ashley Avenue, Charleston, SC 29425 USA

**Keywords:** Prognostic markers, Colonic diseases, Signs and symptoms

## Abstract

**Objective:**

To identify maternal and neonatal risk factors associated with progression to surgery or death after diagnosis of NEC.

**Study design:**

Forty-seven demographic and clinical factors were evaluated across 216 validated cases of NEC occurring between 2010–2020. Nutrition at NEC onset was evaluated in 149 cases. The binary outcome of surgical NEC (progressing to surgery or death) vs. medical NEC (resolved with antibiotic/bowel rest) was compared across variables.

**Results:**

Elevated CRP, rapidly decreasing platelet counts, inotropic medication, intubation, and positive blood cultures within 24 h of diagnosis were associated with progression to surgery/death. Infants with surgical NEC had higher abdominal circumferences at birth. Maternal milk intake and receipt of human milk fortifiers were associated with medical NEC, and infants receiving fortified, maternal milk showed the lowest progression to surgery/death.

**Conclusion:**

The index of suspicion should be heightened for surgical NEC when these risk factors are present.

## Introduction

NEC is a poorly understood process of fulminant inflammation, dysbiosis, and rapid intestinal tissue death representing a leading cause of death in the neonatal intensive care unit [1]. While some NEC cases resolve with nutritional and antibiotic treatment (“medical NEC”), many cases progress to surgical emergencies and/or death (“surgical NEC”) within hours to days of diagnosis. However, there are few consistently reported risk factors, signs, and symptoms that can help a clinician distinguish between medical and surgical NEC upon initial assessment. The goal of this study was to identify novel demographic and clinical factors, both maternal and infant, to help predict a neonate’s likelihood of progressing to surgery or death following NEC diagnosis.

As NEC usually occurs after the onset of enteral feeds, enteral nutrition may represent a critical modifiable factor for NEC severity [2]. While it is well established that maternal milk or “mother’s own milk” is associated with decreased rates of overall NEC, the relationship between nutrition status and NEC severity is not well understood [3–7].

An active area of research is the role of human milk fortifiers (HMF), both bovine- and human-based, in the pathogenesis of NEC. There is concern that the use of bovine-based HMF may increase NEC severity or mortality, but recent studies (including meta-analyses) have concluded no increased risk of NEC with early fortification (before 100 mL/kg/day and before 7 days of life) [8, 9]. Overall mortality (including non-NEC causes) is lower in infants fed human milk-based HMF as compared to bovine-based HMF, though there is no difference in NEC incidence between bovine- and human milk-based HMF [10–12]. This study sought to identify nutritional risk factors that correlate with a neonate’s likelihood of progressing to surgery or death following NEC diagnosis. It was hypothesized that several measurable maternal and perinatal risk factors would be associated with the progression of NEC to surgery and/or death and that human milk fortification would be associated with progression to surgery or death due to NEC.

## Methods

Ten years of patient data were extracted from the Perinatal Information Network System (PINS) from the level IV neonatal intensive care unit (NICU) at the Medical University of South Carolina (MUSC) in Charleston, South Carolina. The Institutional Review Board of MUSC approved this study: Pro00104755 and consent was waived. NEC was defined using Bell’s staging criteria with cases of NEC stage 2A or above, which require a positive radiographic finding of pneumatosis and/or portal venous gas, included. Inclusion was limited to cases occurring between 2010–2020 (*n* = 269). All cases were verified by secondary chart review by a neonatologist or an experienced neonatal nurse practitioner, at which point non-NEC cases with similar clinical presentation (i.e. spontaneous intestinal perforation or SIP), NEC occurring in units other than the NICU, cases transferred in for NEC with original symptoms presenting at an outside hospital, and cases with overall insufficient electronic medical data were excluded for a final cohort (*n* = 216). Forty-seven maternal and neonatal factors were evaluated for each case, including maternal comorbidities, medications, mode of delivery, gestational age, symptoms present at diagnosis, laboratory values before and after diagnosis, and timing between diagnosis and receipt of first antibiotic. The time/date of diagnosis was reported as the time at which the initial blood culture was drawn and used as a proxy for the decision to treat. Cases progressing to surgery or death were classified as “surgical NEC”; cases resolving with antibiotic treatment and bowel rest (cessation of enteral feeds) were classified as “medical NEC.” Mean and standard deviation across variables were reported for both surgical and medical groups. The binary outcome of medical vs. surgical NEC was compared across variables using appropriate student’s t-tests or Pearson’s chi-squared tests. Binary logistic regression was used to adjust for confounding variables such as gestational age, birth weight, antenatal steroids, sex, race, and day of life. The binary outcome of medical vs. surgical NEC was compared across variables similarly using statistical software SPSS v. 28.0. Statistical significance was *p* < 0.05.

To investigate nutritional risk factors, nutrition at the time of NEC diagnosis (contents of singular most recent feed before diagnosis) was characterized categorically. Categories included maternal milk, fortified maternal milk, banked donor human milk, fortified donor human milk, and preterm formula. These categories were not mutually exclusive and babies receiving both maternal milk and formula at the time of NEC (a combined feed before diagnosis) were accounted for within both nutrition categories. Our institution’s fortification guidelines (available in Supplemental Fig. 1) recommend fortification beginning at 100 mL/kg/day or between days 3 and 7 of feeding, depending on the infant’s birth weight. During the period of this study, fortifiers included intact proteins (before 2017) and extensively hydrolyzed protein liquid bovine-based human milk fortifiers (2017 – present) (Similac HMF, Abbott Nutrition, USA).

## Results

A total of 216 verified cases of NEC were analyzed. This included 133 cases of medical NEC (61.6%) and 83 cases of surgical NEC (38.4%). The average gestational age was 29.4 ± 4.2, with birth weight averaging 1290 ± 759 g (Table 1). The medical and surgical groups were comparable in terms of demographics; the only significant demographic differences between groups were a higher prevalence of small for gestational age infants (*p* = 0.038) and a higher age at the time of NEC (*p* = 0.007) in the medical NEC group (Table 1).

**Table 1:** Population demographics for analysis of demographic, clinical, and laboratory characteristics (*n* = 216).

		All NEC (*n* = 216)	Medical (*n* = 133)	Surgical (*n* = 83)	*p*-value
Gestational age (weeks)		29.4 ± 4.2	29.8 ± 4.2	28.7 ± 4.2	0.07
Birth weight (g)		1290 ± 759	1350 ± 758	1202 ± 761	*ns*
Small for gestational age		40 (18.5%)	29 (23%)	11 (14.5%)	0.038
Male		122 (56.5%)	71 (53.4%)	51 (61.4%)	*ns*
Cyanotic heart anomaly present		16 (7.4%)	11 (8.3%)	5 (6.0%)	*ns*
Patent ductus arteriosus		103 (47.7%)	60 (45.1%)	43 (51.8%)	*ns*
Genetic anomaly present		10 (4.6%)	9 (12.9%)	1 (2.4%)	*ns*
APGAR	1 min	4 [2, 7]	4 [2, 7]	4 [2, 7]	*ns*
	5 min	7 [6, 8]	7 [6, 8]	7 [5, 8]	*ns*
Mode of delivery (% vaginal birth)		46 (21.3%)	32 (24.2%)	14 (16.9%)	*ns*
Intrauterine growth restriction (IUGR)		58 (26.9%)	36 (27%)	22 (27%)	*ns*
Race	White	69 (31.9%)	47 (35.3%)	22 (26.5%)	*ns*
	Black	132 (61.1%)	78 (58.6%)	54 (65.1%)	*ns*
	Hispanic	13 (6%)	8 (6%)	5 (6%)	*ns*
	Other	2 (0.9%)	0	2 (2.4%)	*ns*
Maternal antenatal steroids		172 (79.6%)	99 (74.4%)	73 (87.8%)	0.016^a^
Maternal diabetes present		30 (13.9%)	19 (14.1%)	11 (13.3%)	*ns*
Multiple gestations		38 (17.6%)	23 (17.3%)	15 (18.1%)	*ns*
Intra-amniotic infection suspected		13 (6.0%)	10 (7.5%)	3 (3.6%)	*ns*
Maternal tobacco use reported		22 (10.9%)	12 (9.0%)	10 (12.0%)	*ns*
Maternal hypertension		46 (23.1%)	27 (20.3%)	19 (22.9%)	*ns*
Maternal obesity		21 (9.7%)	13 (9.7%)	8 (9.6%)	*ns*
Premature rupture of membranes		39 (18.1%)	29 (21.8%)	10 (12.1%)	0.07
C-section delivery		167 (77.3%)	98 (73.7%)	69 (83.1%)	0.107

Demographics are grouped by outcome of medical NEC (resolved with antibiotics and nil per os) or surgical NEC (progressed to surgery or death). All data is displayed as mean ± standard deviation for continuous variables, median with interquartile ranges [25,75 percentile], or count with percentage in parenthesis. (*p* > 0.05).

*ns* not significant.

^a^Significance remained after adjusting for gestational age and birth weight.

Table 2 demonstrates that the strongest markers predicting the development of surgical NEC were elevated CRP and rapidly decreasing platelets shortly after diagnosis. CRP was higher in cases progressing to surgical NEC at 24 h and 48 h after diagnosis (*p* = 0.022, *p* = 0.002). Platelets were lower in the surgical NEC group within 72 h of diagnosis (*p* < 0.001) and decreased rapidly over the first 24 h post-diagnosis: platelet levels in the surgical group decreased from 208 ± 141 at 24 h to 121 ± 97 k/dL at 48 h (*p* < 0.001). This pattern was not observed in cases of medical NEC, for whom platelet levels did not differ significantly across time points. The surgical NEC group had significantly higher peak and nadir WBC counts within 3 days of diagnosis (*p* = 0.022, *p* = 0.025, Table 2), as well as lower minimum hemoglobin/hematocrit values over this same time frame, (*p* = 0.026, *p* = 0.046, Table 2).

**Table 2:** Clinical and laboratory factors compared between medical and surgical NEC groups.

		Medical (*n* = 133)	Surgical (*n* = 83)	*p*-value
Age at time of NEC diagnosis^b^ (days)		20 [9, 33.5]	13 [7, 25]	0.007
Weight at the time of NEC diagnosis (g)		1768 ± 817	1639 ± 981	*ns*
Abdominal circumference at birth (cm)		22.4 ± 11.1	30.0 ± 26.7	0.032
Maternal antenatal steroids		99 (74.4%)	73 (87.8%)	0.016^a^
Positive blood culture within 24 h of diagnosis		25 (19.1%)	29 (34.9%)	0.009
Pressors within 24 h of diagnosis		6 (4.8%)	56 (71.8%)	<0.001
Intubation within 24 h of diagnosis		17 (13.8%)	45 (55.6%)	<0.001
CRP within 24 h of diagnosis (mg/dL)		4.8 ± 6.0	8.0 ± 5.7	0.022
CRP, 48 h post-diagnosis (mg/dL)		6.6 ± 6.49	13.8 ± 9.36	0.015
Platelets (1000/uL) (time post-diagnosis)	0–24 h	287	208	<0.001
	24–48 h	268	121	
	48–72 h	268	86	
Peak white blood cell count within 5 days of diagnosis (1000 cells/uL)		15.36 ± 6.33	17.92 ± 9.02	0.022
Lowest white blood cell count within 5 days of diagnosis (1000 cells/uL)		4.69 ± 1.73	4.25 ± 4.13	0.025
Lowest hemoglobin ± 3 days of diagnosis (grams/dL)		9.66 ± 1.92	8.92 ± 1.94	0.026
Lowest hematocrit ± 3 days of diagnosis (%)		27.97 ± 5.17	26.77 ± 6.76	0.046
Peak bilirubin ± 3 days of diagnosis (1000 cell/uL)		5.20 ± 4.21	6.35 ± 4.35	*ns*
Antibiotic timing (mins)^c^		100.5 ± 40.8	105.4 ± 90.4	*ns*
Time to full feeds (d)^a^		20.8 ± 17.2	48.4 ± 36.8	0.001
Discharge weight (g)		3252 ± 1321	2657 ± 1804	0.01
Discharge length (cm)		52.1 ± 15.8	47.7 ± 17.0	0.056
Discharge head circumference (cm)		39.5 ± 19.1	39.0 ± 23.2	*ns*

Values are presented as mean ± standard deviation for continuous variables, median with interquartile ranges [25,75 percentile], or count with percentage in parenthesis. (*p* > 0.05).

*ns* not significant.

^a^Time in days between resuming enteral nutrition following NEC diagnosis and reaching a feeding volume of 160 ml/kg/day.

^b^NEC diagnosis documented as date initial blood culture was drawn, used as a proxy for the decision to treat.

^c^Time (minutes) from initial blood culture to time of antibiotic infusion.

Within 24 h following diagnosis, the need for inotropic medications (*p* < 0.001), intubation (*p* < 0.001), or positive blood culture results (*p* = 0.009) were associated with an increased risk of progression to surgery and/or death. Of 62 medical and surgical NEC cases with positive blood cultures taken within 24 h of diagnosis, the predominant species were coagulase-negative *Staphylococcus* (27.4%), *E. coli* (22.6%), and *Klebsiella* species (12.9%). Abdominal circumference at birth was significantly higher in the surgical NEC group (mean 30.0 ± 26.7 cm) as compared to our medical NEC group (mean 22.4 ± 11.1 cm; *p* = 0.032). The use of maternal steroids was associated with an increased prevalence of progression to surgical NEC or death (*p* = 0.016). This significance remained after adjustment for gestational age and birth weight. Infants who developed surgical NEC took significantly longer to progress to full feeds post-NEC (*p* = 0.001) and had lower discharge weights (*p* = 0.010) and lengths (*p* = 0.056) than did medical NEC patients. A significant difference was not observed in factors such as neonatal birth weight, gestational age, mode of delivery, antibiotic timing, or APGAR score.

One hundred forty-nine NEC cases had electronic medical records with neonatal nutrition data available at the time of NEC diagnosis. This included 100 cases of medical NEC (67%) and 49 cases of surgical NEC (33%). Of these cases, the average gestational age was 29.8 ± 4.4 weeks with birth weight averaging 1386 ± 802 g (Table 3). The medical NEC group had a higher age at the time of NEC (20.5 days [11–34]) compared to the surgical NEC group (11 days [6–23]; *p* = 0.004). There were no significant differences between gestational age, birth weight, or weight at the time of NEC diagnosis between medical and surgical NEC groups.

**Table 3:** Population demographics with nutritional analysis (*n* = 149).

		NEC with nutrition data available (*n* = 149)
Gestational age (weeks)		29.8 ± 4.4 weeks
Birth weight (g)		1386 ± 802 g
Age at the time of NEC diagnosis (days)		18 [9–31] days
Weight at the time of NEC diagnosis (g)		1719 ± 848 g
Medical NEC (resolved with antibiotics and NPO)		100 (67%)
Surgical NEC (progressed to surgery or death)		49 (33%)
Nutrition at the time of NEC	Maternal milk	46 (31%)
	Fortified maternal milk	64 (43%)
	Donor milk	21 (14%)
	Fortified donor milk	14 (9%)
	Formula	41 (28%)
	Total receiving fortification	72 (61%)
	Total receiving any maternal milk	122 (82%)

Values are presented as mean ± standard deviation for continuous variables, median with interquartile ranges [25, 75 percentile], or count with percentage in parenthesis.

**Fig. 1:**
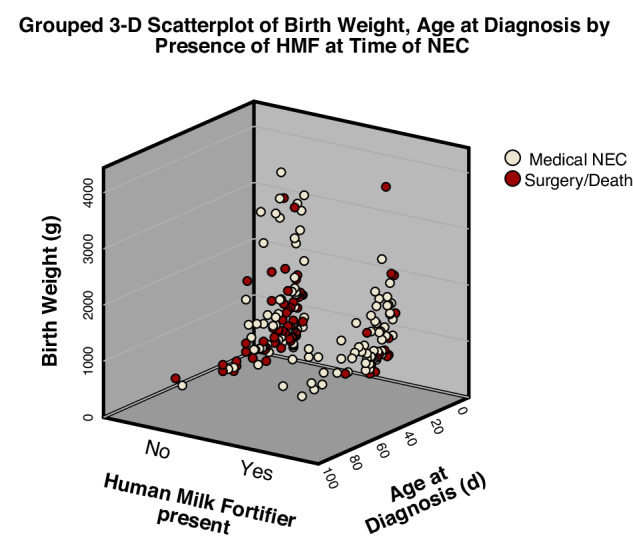
Grouped  3-dimensional scatter plot of medical and surgical NEC cases by fortification status, age, and birth weight. Dot colors indicate severity (red = surgical/death, white = medical). Severe NEC is more prevalent in the “No Human Milk Fortifier present” group than the group receiving fortifier immediately before diagnosis, and the cases are similarly distributed by birthweight and age of diagnosis.

Infants fed with maternal milk at the time of NEC development showed a lower likelihood of progressing to surgical NEC, though this result was not significant (*p* = 0.160, Table 4). Fortification of any human milk (maternal or donor) was associated with significantly fewer cases of surgical NEC (*p* = 0.01; Fig. 1). Neonates receiving fortified maternal milk showed a significantly lower prevalence of surgical NEC than babies fed with any other nutrition type (*p* = 0.033). We did not find any significant difference in formula use between the medical and surgical NEC groups (*p* = 0.330).

**Table 4:** Nutrition at the time of NEC diagnosis (characterized by last feed preceding diagnosis) for medical and surgical NEC groups, respectively.

Nutrition type received before the time of NEC diagnosis^a^	Medical NEC (*n* = 100)	Surgical NEC (*n* = 49)	*p*-value
Any fortified human milk	56 (68.3%)	16 (43.2%)	0.01
Any non-fortified human milk	26 (31.7%)	21 (56.8%)	
Fortified maternal milk	49 (49%)	15 (30.6%)	0.033
Other categories (non-fortified maternal milk, any donor milk, or formula)	51 (51%)	34 (69.4%)	
Any maternal milk	85 (85%)	37 (75.5%)	0.16
Non-maternal milk and formula	15 (15%)	12 (24.5%)	
Total receiving formula	25 (25%)	16 (32.7%)	0.33
Total receiving any human milk (donor or maternal)	75 (75%)	33 (67.3%)	

Significance assesses for differences in proportion (%) receiving that nutrition type in the medical vs. surgical group.

^a^Documentation of feeding type immediately before NEC diagnosis. The fortification used was a liquid bovine-based human milk fortifier. Values are presented as counts with percentages in parentheses. Significance is *p* < 0.05.

## Discussion

This cohort exhibited several clinical and laboratory markers associated with an increased risk of progression to surgery or death following NEC diagnosis, many of which corroborate existing studies. Unlike the medical NEC group, the surgical NEC group exhibited significantly lower platelet levels at 24-, 48-, and 72 h post-diagnosis, as well as a significant drop in values between 24 and 48 h. This finding is consistent with previous literature, which demonstrates that a lower platelet count is associated with a greater extent of intestinal deterioration and that platelets are lower in infants who die from NEC than in survivors [13, 14].

Prior research has demonstrated elevated CRP is a biomarker for NEC diagnosis and elevated CRP concentrations are associated with higher risk of surgery [15, 16]. The CRP levels of the surgical NEC group were consistent with this trend, with a significant and rapid rise in CRP between 24 and 48 h post-diagnosis. CRP has been previously shown to be an indicator of antibiotic responsiveness, thus increasing CRP concentrations in the setting of surgical NEC suggests a failure to respond to initial antibiotic treatment [17]. While CRP is nonspecific and cannot be used to exclude or confirm a diagnosis of NEC, it has utility as a marker of disease progression in known cases of NEC. Other significant laboratory differences included both peak and nadir WBC counts within 3 days of diagnosis, with surgical NEC showing higher peak WBC counts and lower WBC nadirs. Also, surgical NEC was associated with lower hemoglobin and hematocrit levels proximal to the time of diagnosis. These findings reflect the critical illness associated with surgical NEC as compared to medical NEC. There were no differences in peak bilirubin levels taken proximal to the time of diagnosis.

A positive blood culture and the need for vasopressors or intubation within 24 h of diagnosis were also associated with NEC severity, supporting prior similar findings [14]. When taken together, the above laboratory and clinical indicators may be used to assess an infant’s likelihood of progressing to surgery or death at the time of diagnosis and “flag” high-risk cases for increased monitoring and lower clinical threshold for surgical intervention.

Consistent with the standing literature, intake of any maternal milk was associated with medical NEC vs. surgical NEC in this cohort, though this result was not significant. Babies receiving fortification of any human milk also showed significantly less progression to surgery or death than babies receiving unfortified milk. This association remained even after adjustments for age of diagnosis and birth weight, meaning this finding was not associated with the timing of the feeding protocol. Clinical events prompting medical providers to remove fortifiers may contribute to this relationship. For example, the perceived reaction to fortification and subsequent removal of HMF may be a risk factor for the development of severe NEC. Feeding type (mother’s milk vs. formula) has been shown to alter splanchnic intestinal blood flow in neonates [18]. It is unknown if the increasing caloric density of nutrition affects splanchnic blood flow or tissue oxygenation during digestion. Feeding intolerance may serve to unmask other hemodynamic issues such as a patent ductus arteriosus. However, the removal of fortification (or withholding fortification) did not seem to protect neonates against the development of severe NEC in this cohort, as many of the severe NEC cases were not receiving fortification at the time of diagnosis (Fig. 1).

Alternatively, fortification may be protective against severe NEC. Prior studies have demonstrated similar or reduced rates of NEC with earlier receipt of human milk fortification, though the overall relationship is not well understood [6, 10, 19]. Overall, these findings support the idea that early fortification and strict adherence to feeding protocols may help reduce cases of NEC-related surgery and death [20].

This study did not find a significant difference in NEC severity between infants fed with formula versus human milk, though an increased incidence of NEC in formula-fed neonates has been extensively observed [3, 7, 21]. The majority of infants included in this study were premature and received either donor human milk or maternal milk by protocol, therefore this study was not large enough to observe this effect. As a follow-up to a previous pilot study, this data did not show a significant correlation between NEC severity and the time between NEC diagnosis and antibiotic initiation [22].

This study identified a novel risk factor in the development of severe NEC: abdominal circumference at birth was significantly larger in the surgical NEC group, even though these neonates trended towards having lower birth weights (Table 1). This suggests that increased abdominal circumference at birth may be associated with a higher risk of progressing to surgery or death following NEC diagnosis. This could signify early-onset intestinal immaturity and underlying motility issues in these infants, predisposing these infants to more severe GI outcomes. Abdominal distention is a common presenting clinical sign preceding NEC diagnosis, though NEC did not occur until the mean day of life 29.4 in this cohort; therefore, this marker could be detected approximately 30 days before full disease onset. Interestingly, a study found that preterm infants with a higher risk of NEC had altered superior mesenteric artery dopplers on day of life 1 [23]. Therefore, distension may be related to poor motility or a consequence of intestinal blood flow. Of note, many hospitals nationwide have recently discontinued the measurements of abdominal circumference in their standard anthropometric birth measurements. A limitation of this observation includes the evaluation of positive pressure support or ventilation at delivery. Delivery interventions were not analyzed in this study. Further research may be warranted to confirm the significance of this measurement as a risk factor for severe NEC.

In this cohort, there was a higher proportion of neonates who received antenatal steroids (ANS) in the surgical NEC group as compared to the medical NEC group. This could be an effect of ANS being more often administered to smaller and more premature neonates; however, the relationship and its significance remained after adjustment for gestational age and birth weight. This finding was initially surprising as multiple sources, including a 2017 Cochrane meta-analysis report, lower the overall incidence of NEC with receipt of ANS [7, 24]. However, the association between ANS use and NEC severity is not well studied, and other studies report similar findings to those seen in this cohort. Chawla et al. released a cohort study in 2022 reporting higher rates of medical NEC, 12.3% vs. 6.9% (OR 1.30, 95% CI 0.56–3.04), and higher rates of surgical NEC, 6.6% vs. 4.2% (OR = 2.05; 95% CI 0.57–7.35), among neonates receiving a complete course of antenatal steroids between 21 and 22 weeks gestation as compared with those receiving partial or no steroids, though there was a lower rate of all-cause death (including that from surgical NEC) among the group receiving a complete course of steroids (OR 0.69; 95% CI 0.42–1.15) [25]. Though their results regarding NEC did not reach significance, they showed the same trends seen in this study (i.e. lower rates of surgical NEC with increased ANS receipt) throughout all of their comparisons. Other studies report neither a risk nor protective relationship between ANS receipt and NEC rates, though ANS receipt does lower overall neonatal mortality [26, 27]. More work is needed to assess the true relationship between antenatal steroid use and NEC severity, which is likely complex and multifactorial.

This study included neonates with cyanotic heart disease and patent ductus arteriosus (PDA) in its analyses. Cardiac NEC is suggested to be a distinct disease due to differences in initial severity, age at diagnosis, and prognoses between subjects with necrotizing enterocolitis with and without heart disease [28]. Thus, it was necessary to assess for any differences in neonatal cardiac anomalies between the medical and surgical NEC groups. There was no significant difference in cyanotic heart disease (8.3% in medical NEC, 6.0% in surgical NEC, *p* = 0.53) or PDA (45.1% in medical NEC, 51.8% in surgical NEC, *p* = 0.34) between the two outcome groups (see Table 1). This suggests that cardiac NEC within this cohort did correlate with the outcome of surgery or death. Additionally, as rates are highly similar between the two groups, cardiac NEC is unlikely to have influenced other differences seen between outcome groups. The study is however limited in that it did not distinguish between cardiac and non-cardiac NEC, and so cannot draw conclusions about whether the demographic, laboratory, clinical, or nutrition factors discussed are more specific to one subtype of NEC or the other.

Additional limitations of this study include the fact that CRP in the surgical group may be confounded by the surgery itself; i.e. some of the elevated CRP levels occurring within 72 h post-diagnosis in the surgical group occurred following neonatal surgery and therefore could be a result of the operation itself rather than an indicator of disease severity. However, of the 36 surgical NEC cases that exhibited a CRP value above 0.07 mg/dL (above the upper limit of normal), only 8 of these cases had CRP values included in this study which occurred following surgical intervention, and four of the eight had already displayed high (significantly >0.07 mg/dL) CRP levels before any surgical intervention. Overall, the influence of surgical intervention on CRP data is unlikely to have affected overall data trends but should be accounted for when applying this finding to clinical practice.

This study’s characterization of nutrition using the single most recent feed before NEC diagnosis also presents a limitation, as this method provides only a snapshot of nutrition status and may not be wholly reflective of the neonate’s diet. For example, the infant may have been receiving maternal milk for several weeks but was supplemented with a donor milk feed as their last feed before NEC diagnosis, and so was categorized as a “donor milk” case in our study. Future research assessing a wider data set of feedings for each neonate is required to assess the validity of the data presented here.

Additionally, thrombocytopenia, one of the significant markers of progression to surgery/death in this study, is included as a sign in the modified Bell Staging criteria for NEC. As the modified Bell staging criteria are known and used widely, the presence of this laboratory anomaly included in the Bell criteria may have influenced clinicians’ decisions for surgical management, confounding the association between thrombocytopenia and surgical NEC. Finally, this study does not account for populations without a diagnosis of NEC, so the risk factors described are limited by this perspective. However, as this research corroborated well-studied associations relating NEC to elevated CRP, decreasing platelet count, and lower maternal milk intake, there is established validity to the findings. The study only accounts for associations between risk factors and NEC outcomes and does not conclude causal relationships.

In summary, elevated CRP, low platelets, positive blood culture results, and the need for inotropic medication or vasopressors within 24 h of NEC diagnosis are significantly associated with progression to NEC-related surgery or death. Few maternal or demographic markers predicted the risk of surgical vs. medical NEC beyond birth weight and gestational age. Neonates receiving fortified human milk showed lower rates of NEC-related surgery or death; surgery and death rates were lowest among neonates receiving fortified, maternal milk compared to all other nutrition types. Increased abdominal circumference at birth is a potential early marker of increased risk for severe NEC. Further investigation is warranted to clarify the association between antenatal steroid receipt and NEC severity and to determine the utility above markers in clinical practice.

## Supplementary information

Supplemental Material Figure Legend

Supplemental Material Figure

## Data Availability

The datasets without patient identifiers analyzed during the current study are available from the corresponding author upon reasonable request.

## References

[1] Patel RM, Kandefer S, Walsh MC, Bell EF, Carlo WA, Laptook AR, et al. Causes and timing of death in extremely premature infants from 2000 through 2011. N Engl J Med. 2015;372:331–40. 25607427 10.1056/NEJMoa1403489 PMC4349362

[2] Abrams SA, Schanler RJ, Lee ML, Rechtman DJ. Greater mortality and morbidity in extremely preterm infants fed a diet containing cow milk protein products. Breastfeed Med. 2014;9:281–5. 24867268 10.1089/bfm.2014.0024 PMC4074755

[3] Lucas A, Cole TJ. Breast milk and neonatal necrotising enterocolitis. Lancet. 1990;336:1519–23. 1979363 10.1016/0140-6736(90)93304-8

[4] Meinzen-Derr J, Poindexter B, Wrage L, Morrow A, Stoll B, Donovan E. Role of human milk in extremely low birth weight infants’ risk of necrotizing enterocolitis or death. J Perinatol. 2009;29:57–62. 18716628 10.1038/jp.2008.117 PMC2801431

[5] Patel AL, Kim JH. Human milk and necrotizing enterocolitis. Semin Pediatr Surg. 2018;27:34–38. 29275815 10.1053/j.sempedsurg.2017.11.007

[6] Schanler RJ, Shulman RJ, Lau C. Feeding strategies for premature infants: beneficial outcomes of feeding fortified human milk versus preterm formula. Pediatrics. 1999;103:1150–7. 10353922 10.1542/peds.103.6.1150

[7] Rose AT, Patel RM. A critical analysis of risk factors for necrotizing enterocolitis. Semin Fetal Neonatal Med. 2018;23:374–9. 10.1016/j.siny.2018.07.005 PMC6269219 30115546

[8] Basu S, Upadhyay J, Singh P, Kumar M. Early versus late fortification of breast milk in preterm infants: a systematic review and meta-analysis. Eur J Pediatrics. 2020;179:1057–68. 10.1007/s00431-020-03677-6 32458060

[9] Alyahya W, Simpson J, Garcia AL, Mactier H, Edwards CA. Early versus delayed fortification of human milk in preterm infants: a systematic review. Neonatology. 2020;117:24–32. 31326969 10.1159/000501279

[10] O’Connor DL, Kiss A, Tomlinson C, Bando N, Bayliss A, Douglas C, et al. Nutrient enrichment of human milk with human and bovine milk–based fortifiers for infants born weighing< 1250 g: a randomized clinical trial. Am J Clin Nutr. 2018;108:108–16. 29878061 10.1093/ajcn/nqy067

[11] Jensen GB, Domellöf M, Ahlsson F, Elfvin A, Navér L, Abrahamsson T. Effect of human milk-based fortification in extremely preterm infants fed exclusively with breast milk: a randomised controlled trial. eClinicalMedicine. 2024;68:102375. 38545091 10.1016/j.eclinm.2023.102375 PMC10965410

[12] Galis R, Trif P, Mudura D, Mazela J, Daly MC, Kramer BW, et al. Association of Fortification with Human Milk versus Bovine Milk-Based Fortifiers on Short-Term Outcomes in Preterm Infants—A Meta-Analysis. Nutrients. 2024;16:910. 38542821 10.3390/nu16060910 PMC10975992

[13] Ververidis M, Kiely E, Spitz L, Drake D, Eaton S, Pierro A. The clinical significance of thrombocytopenia in neonates with necrotizing enterocolitis. J Pediatr Surg. 2001;36:799–803. 11329593 10.1053/jpsu.2001.22964

[14] Garg PM, Paschal JL, Ansari MAY, Block D, Inagaki K, Weitkamp J-H. Clinical impact of NEC-associated sepsis on outcomes in preterm infants. Pediatr Res. 2022;92:1705–15. 35352003 10.1038/s41390-022-02034-7 PMC10311923

[15] Yu D, Yang H, Zhong C, Fan K, Zeng G, Zhang Z, et al. Pneumonia, lymphocytes and C-reactive protein are valuable tests for predicting surgical intervention in necrotizing enterocolitis. Front Pediatrics. 2023;11:1231627. 10.3389/fped.2023.1231627 PMC10419212 37576139

[16] Mohd Amin AT, Zaki RA, Friedmacher F, Sharif SP. C-reactive protein/albumin ratio is a prognostic indicator for predicting surgical intervention and mortality in neonates with necrotizing enterocolitis. Pediatr Surg Int. 2021;37:881–6. 33779823 10.1007/s00383-021-04879-1 PMC8005510

[17] Dias RF, De Paula AC, Hasparyk UG, Rabelo M, Alderete JR, Chihondo JK, et al. Use of C-reactive protein to guide the antibiotic therapy in hospitalized patients: a systematic review and meta-analysis. BMC Infect Dis. 2023;23:276. 37138222 10.1186/s12879-023-08255-3 PMC10155296

[18] Coombs R, Morgan M, Durbin G, Booth I, McNeish A. Doppler assessment of human neonatal gut blood flow velocities: postnatal adaptation and response to feeds. J Pediatr Gastroenterol Nutr. 1992;15:6–12. 1403452 10.1097/00005176-199207000-00002

[19] Hair AB, Scottoline B, Good M. Dilemmas in human milk fortification. J Perinatol. 2023;43:103–7. 36097287 10.1038/s41372-022-01502-6 PMC10317058

[20] Jasani B, Patole S. Standardized feeding regimen for reducing necrotizing enterocolitis in preterm infants: an updated systematic review. J Perinatol. 2017;37:827–33. 28358382 10.1038/jp.2017.37

[21] Schanler RJ, Lau C, Hurst NM, Smith EOB. Randomized trial of donor human milk versus preterm formula as substitutes for mothers’ own milk in the feeding of extremely premature infants. Pediatrics. 2005;116:400–6. 16061595 10.1542/peds.2004-1974

[22] Chetta KE, Vincent KG, Fanning B, Klumb AB, Chetta J, Rohrer A, et al. Impact of Delayed Time to Antibiotics in Medical and Surgical Necrotizing Enterocolitis. Children. 2023;10:160. 36670710 10.3390/children10010160 PMC9856867

[23] Murdoch EM, Sinha AK, Shanmugalingam ST, Smith GC, Kempley ST. Doppler flow velocimetry in the superior mesenteric artery on the first day of life in preterm infants and the risk of neonatal necrotizing enterocolitis. Pediatrics. 2006;118:1999–2003. 17079572 10.1542/peds.2006-0272

[24] Roberts D. Antenatal corticosteroids for accelerating fetal lung maturation for women at risk of preterm birth. *Cochrane Database Syst Rev*. 2007;4:CD004454. 10.1002/14651858.CD004454.pub2 16856047

[25] Chawla S, Wyckoff MH, Rysavy MA, Patel RM, Chowdhury D, Natarajan G, et al. Association of antenatal steroid exposure at 21 to 22 weeks of gestation with neonatal survival and survival without morbidities. JAMA Netw Open. 2022;5:e2233331. 36156145 10.1001/jamanetworkopen.2022.33331 PMC9513645

[26] Lamireau N, Greiner E, Hascoët J-M. Risk factors associated with necrotizing enterocolitis in preterm infants: A case–control study. Arch de Pédiatrie. 2023;30:477–82. 10.1016/j.arcped.2023.07.003 37704519

[27] Neu J, Pammi M. Necrotizing enterocolitis: The intestinal microbiome, metabolome and inflammatory mediators. Semin Fetal Neonatal Med. 2018;23:400–5. 30172660 10.1016/j.siny.2018.08.001

[28] Burge KY, Gunasekaran A, Makoni MM, Mir AM, Burkhart HM, Chaaban H. Clinical characteristics and potential pathogenesis of cardiac necrotizing enterocolitis in neonates with congenital heart disease: a narrative review. J Clin Med. 2022;11:3987. 35887751 10.3390/jcm11143987 PMC9320426

