# Supplementary material for: Maternal, neonatal, and nutritional risk factors for medical and surgical necrotizing enterocolitis
Source: J Perinatol. 2024 Jul 19;44(12):1762–7. doi: 10.1038/s41372-024-02066-3 (PMC11606919; doi:10.1038/s41372-024-02066-3)
Supplement: Supplementary file 1 — Supplemental Material Figure Legend [file 41372_2024_2066_MOESM1_ESM.docx]

**Supplemental Figure 1**: Fortification Guidelines at the Laura and David Stone Neonatal Intensive Care Unit at the Medical University of South Carolina
