# Supplementary material for: Maternal, neonatal, and nutritional risk factors for medical and surgical necrotizing enterocolitis
Source: J Perinatol. 2024 Jul 19;44(12):1762–7. doi: 10.1038/s41372-024-02066-3 (PMC11606919; doi:10.1038/s41372-024-02066-3)
Supplement: Supplementary file 2 — Supplemental Material Figure [file 41372_2024_2066_MOESM2_ESM.pdf]

MUSC NICU Fortification Guidelines

- Volume in mL/kg per feeding protocol day:

	Day 0		Day 1		Day 2		Day 3		Day 4		Day 5		Day 6		Day 7		Day 8		Day 9		Day 10	
Weight (g)	*AM*	*PM*	*AM*	*PM*	*AM*	*PM*	*AM*	*PM*	*AM*	*PM*	*AM*	*PM*	*AM*	*PM*	*AM*	*PM*	*AM*	*PM*	*AM*	*PM*	*AM*	*PM*
**<1000**	**12**		**12**		**12**		**12**		**24**	**36**	**48**	**60**	**72**	**88**	**100**	**100**	**112**	**124**	**136**	**152**	**160**	**160**
**1000 – 1499**	**12**		**12**		**28**	**40**	**56**	**72**	**88**	**100**	**100**	**120**	**132**	**144**	**160**	**160**						
**≥1500**	**32**		**32**		**48**	**64**	**84**	**100**	**100**	**120**	**136**	**152**	**160**	**160**								

= Fortification with Similac Bovine Human Milk Fortifier, hydrolyzed protein to **24 kcal/oz**
